# Adjuvant Radiotherapy in Surgically Treated HPV-Positive Oropharyngeal Carcinoma with Adverse Pathological Features

**DOI:** 10.3390/cancers14184515

**Published:** 2022-09-17

**Authors:** Shady I. Soliman, Farhoud Faraji, John Pang, Loren K. Mell, Joseph A. Califano, Ryan K. Orosco

**Affiliations:** 1 School of Medicine, University of California San Diego, La Jolla, CA 92093, USA; 2 Department of Otolaryngology-Head & Neck Surgery, University of California San Diego, La Jolla, CA 92037, USA; 3 Department of Otolaryngology-Head & Neck Surgery, Louisiana State University, Shreveport, LA 71103, USA; 4 Department of Radiation Medicine and Applied Sciences, University of California San Diego, La Jolla, CA 92037, USA; 5 Moores Cancer Center, La Jolla, CA 92037, USA

**Keywords:** adjuvant radiotherapy, HPV-positive oropharyngeal cancer, adverse features, positive margins, extranodal extension, lymphovascular invasion, level 4/5 cervical lymph nodes, de-intensification

## Abstract

**Simple Summary:**

Human papillomavirus-positive oropharyngeal carcinoma (HPV-OPC) is being increasingly treated with upfront surgery. Whether patients require further “adjuvant” treatment, such as radiation, depends on microscopic “adverse features” identified on pathological analysis of the resected tumor specimen. Current guidelines recommend adjuvant radiotherapy for HPV-OPC tumors that demonstrate adverse features. In the present study, we demonstrate that adjuvant radiotherapy is associated with improved overall survival in patients with early-stage HPV-OPC who were found to have adverse pathological features. However, the rate of patients with adverse feature positive HPV-OPC who did not receive adjuvant radiotherapy significantly increased during the study period, from 10% in 2010 to 17% in 2017.

**Abstract:**

Purpose: HPV-positive oropharyngeal carcinoma (HPV-OPC) is increasingly treated with primary surgery. The National Comprehensive Cancer Network (NCCN) recommends adjuvant therapy for surgically treated HPV-OPC displaying adverse pathological features (AF). We evaluated adjuvant radiotherapy patterns and outcomes in surgically treated AF-positive HPV-OPC (AF-HPV-OPC). Methods: The National Cancer Database was interrogated for patients ≥ 18 years with early-stage HPV-OPC from 2010 to 2017 who underwent definitive resection. Patients that had an NCCN-defined AF indication for adjuvant radiotherapy were assessed, including positive surgical margins (PSM), extranodal extension (ENE), lymphovascular invasion, and level 4/5 cervical lymph nodes. Overall survival (OS) was evaluated using Cox proportional hazards models and Kaplan–Meier analysis in whole and propensity score matched (PM) cohorts. Results: Of 15,036 patients meeting inclusion criteria, 55.7% were positive for at least one AF. Presence of any AF was associated with worse OS (hazard ratio (HR) = 1.56, *p* < 0.001). In isolation, each AF was associated with worse OS. On PM analysis, insurance status, T2 category, Charlson-Deyo comorbidity score, ENE (HR = 1.81, *p* < 0.001), and PSM (HR = 1.58, *p* = 0.002) were associated with worse OS. Median 3-year OS was 92.0% among AF-HPV-OPC patients undergoing adjuvant radiotherapy and 84.2% for those who did not receive adjuvant radiotherapy (*p* < 0.001, n = 1678). The overall rate of patients with AF-HPV-OPC who did not receive adjuvant radiotherapy was 13% and increased from 10% in 2010 to 17% in 2017 (p_trend_ = 0.007). Conclusions: In patients with AF-HPV-OPC, adjuvant radiotherapy is associated with improved survival. In the era of de-escalation therapy for HPV-OPC, our findings demonstrate the persistent prognostic benefit of post-operative radiotherapy in the setting of commonly identified adverse features. Ongoing clinical trials will better elucidate optimized patient selection for de-escalated therapy.

## 1. Introduction

Human papillomavirus-positive oropharyngeal carcinoma (HPV-OPC) continues to increase in incidence globally [1] and currently accounts for over 70% of all oropharyngeal squamous cell carcinomas in the United States [2,3]. Patients with HPV-OPC display a favorable prognosis compared to those with HPV-negative OPC [4,5]. As a result, therapeutic goals for patients with HPV-OPC have increasingly incorporated measures to not only achieve excellent oncologic outcomes, but to also mitigate therapeutic morbidities in an effort to enhance quality of life. The development and modern refinement of transoral surgical techniques has bolstered the armamentarium of curative-intent therapies for HPV-OPC. There has been increasing adoption of surgery as a primary treatment modality of HPV-OPC and promising potential to reduce treatment-related morbidities [6,7]. Transoral surgery has been incorporated into prospective therapeutic deintensification trials [8,9,10], and patient selection for deintensification remains an area of active investigation.

Surgical extirpation enables the histopathological evaluation of resected specimens for factors associated with poor prognosis, known as adverse pathological features (AF). In the context of head and neck carcinoma, AF include positive surgical tumor margins (PSM), perineural invasion (PNI), lymphovascular invasion (LVI), extranodal extension (ENE), or level 4/5 positive cervical lymph nodes (LN4/5). PSM indicate incomplete tumor resection, the presence of tumor in the surgical bed, and represent a risk factor for tumor recurrence and metastasis [11]. PNI, LVI, ENE, and LN4/5 are microscopic indicators of a more aggressive disease phenotype that is disproportionately invasive, prone to metastasis, and therapeutic resistance [12,13].

Considering that 15–20% of patients with HPV-OPC experience disease recurrence [4,5], debate has emerged regarding patient selection for adjuvant radiotherapy in surgically treated HPV-OPC. Retrospective studies have revealed that adverse features in HPV-OPC may be associated with worse overall survival (OS) [14,15]. The National Comprehensive Cancer Network (NCCN) recommends adjuvant therapy for surgically treated HPV-OPC demonstrating AF [16]. However, the prognostic effect of NCCN-designated adverse features is incompletely understood in the context of HPV-OPC. Data determining the pathologic features that require adjuvant therapy is largely based on HPV-negative disease [17,18]. Studies have investigated which adverse features require further management with adjuvant therapy in surgically treated HPV-OPC [19,20,21,22]. However, the individual role of each adverse feature on survival [15] and whether adjuvant radiotherapy improves survival in patients with adverse feature positive HPV-OPC is not well defined, especially for patients with early-stage disease that is amenable for primary surgical treatment. Taking these insights into account, in the present study we aimed to evaluate the individual prognostic effect of specific AF in HPV-OPC and to understand practice patterns for adjuvant radiotherapy and their associated outcomes in surgically treated AF-positive HPV-OPC (AF-HPV-OPC).

## 2. Materials and Methods

### 2.1. Patient Population

The NCDB is a nationwide database that includes oncologic outcomes from >1500 Commission on Cancer-accredited facilities and includes information on over 70% of new cancer diagnoses in the United States. Patient data were analyzed from the NCDB version 2018. Patients ≥ 18 years with HPV-positive disease of the oropharynx diagnosed between January 1, 2010, and December 31, 2017 with American Joint Committee on Cancer TNM stage I-II disease were included (n = 46,469). Patients with unknown or T3 and T4 category disease (n = 14,492), or not treated with primary surgery (n = 16,346), with unknown adjuvant radiotherapy status (n = 521), or unknown adverse feature status (n = 74) were excluded (Figure S1). This study was deemed exempt from review by the University of California San Diego Institutional Review Board.

### 2.2. Variable Definitions

Patients diagnosed with squamous cell carcinoma arising from the oropharynx primary site were identified based on International Classification of Diseases for Oncology, Third Edition (ICD-O-3) codes. Oropharynx was defined as C019, C024, C051, C052, C090, C091, C098-C100, C102-104, C108, C109, or C142 [23]. Squamous cell histology was based on codes 8052, 8070–8076, 8078, 8083. HPV-negative OPCs were classified as HPV-negative for both high and low-risk types and HPV-positive for low-risk types only (codes 000 and 010). HPV-positivity was defined as tumors with any high-risk serotype or HPV-positive tumors with unspecified risk or type (codes 020-070) [24]. Included patients were tumor positive for HPV and underwent definitive primary surgery. TNM staging was based on pathologic tumor and nodal categories except for tumors lacking pathologic categorization, which were coded based on clinical tumor (n = 1551) and nodal categories (n = 3657). Variables included in our analysis were age, sex, race, Charlson-Deyo Comorbidity score, primary payor, median household income, and adverse features.

The NCDB estimates median household income for each patient’s area of residence by matching the zip code of the patient recorded at the time of diagnosis against files derived from the American Community Survey data, adjusting for inflation. Household income is categorized as quartiles based on equally proportioned income ranges among all US zip codes. Median household income by quartile is as follows: lowest quartile: less than $40,227, second quartile: $40,227–$50,353, third quartile: 3 $50,354–$63,332, upper quartile: ≥ $63,333. Please see the NCDB PUF Data Dictionary 2019 and https://www.census.gov/acs/ (accessed on 6 September 2022) for more information.

AF analyzed included macroscopic or microscopic residual tumor at the primary site (positive surgical margins), macroscopic or microscopic tumor extension beyond the lymph node capsule (extracapsular extension), presence of tumor cells in level 4/5 or retropharyngeal lymph nodes, and microscopic presence of tumor cells in lymphatics or blood vessels (lymphovascular invasion). Perineural invasion was not included in this analysis because it was not coded in NCDB. All AJCC 6th and 7th edition tumor and nodal categories in patients from 2010–2017 were recoded to AJCC 8th edition [25,26].

### 2.3. Statistical Analysis

Descriptive analyses were performed for patient demographics and tumor characteristics using 2-sample Student’s t-test and Pearson’s chi-square tests for continuous and categorical variables, respectively. Pearson correlation correlograms were performed for each AF pair in whole and propensity score matched cohorts. Unadjusted and adjusted Cox proportional hazard analyses were performed to evaluate survival-time formatted data to determine hazard ratios (HR) and 95% confidence intervals (CI). Analyses were adjusted for variables found to be statistically significant. Overall survival (OS) was calculated based on the date of diagnosis. Survival curves were plotted by Kaplan–Meier method and OS rates were compared using log-rank test. To account for differences in covariates among patients who received adjuvant radiotherapy for AF-HPV-OPC, factors associated with survival on unadjusted Cox analyses were included for propensity score matching. Propensity score matching for AF-HPV-OPC who received adjuvant radiotherapy (Rad+) versus no adjuvant radiotherapy (Rad−) was performed using a propensity score matching algorithm with a caliper width of 0.02 [27]. To address potential selection bias, unadjusted and adjusted Cox proportional hazard analyses and Kaplan–Meier analyses were applied to propensity score matched cohorts. Stata/IC version 28.0 (StataCorp, College Station, TX, USA) was utilized for statistical analysis. The alpha level for statistical significance was set at 0.05.

## 3. Results

### 3.1. Baseline Characteristics

The analytic cohort consisted of 15,036 patients. Consistent with clinicodemographic characteristics reported for HPV-OPC [28], most patients were male (83.6%) and of white race (91.7%). Mean age of the full cohort was 58.3 years (standard deviation [SD] = 9.4). Demographic and clinical characteristics are detailed in Table 1.

**Figure 1:**
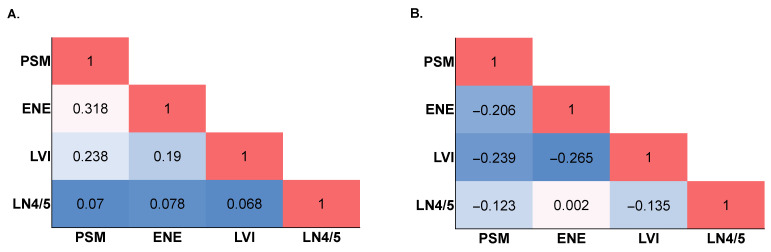
AF Correlogram (**A**) AF Correlogram in HPV-OPC Cohort (**B**) AF Correlogram in PM Cohort.

Of the total population of patients with HPV-OPC, 8375 (55.4%) were positive for at least one AF, 6661 (44%) were negative for any AF, and 74 (0.5%) had unknown AF status. More AF-positive patients were male (84.5% vs. 82.3%, *p* < 0.001), had palatine tonsil primary (72.6% vs. 70.4%, *p* = 0.002), had more advanced primary tumor category (T2: 48.4% vs. 43%, *p* < 0.001), and more advanced lymph node disease (N3: 4.1% vs. 1.5%, *p* < 0.001). No significant difference was observed in comorbidity score with regard to AF status (*p* = 0.107) (Table 1). In the analytic cohort, tumors with any individual AF were positively correlated with having co-localization of other AFs (Figure 1A).

Of AF-HPV-OPC patients, 4033 (48.2%) had PSM, 2809 (33.5%) had LVI, 3238 (38.7%) had ENE, and 1710 (20.4%) had LN4/5 (Table 1). Patients with AF-HPV-OPC were more likely to receive adjuvant radiotherapy (86.8% vs. 68.1%, *p* < 0.001). Younger patients (mean age 57.9 vs. 60.9 years, *p* < 0.001), those with primary tumors of the palatine tonsil (73.8% vs. 64.4%, *p* < 0.001), more advanced lymph node disease (N1: 82% vs. 71.9%, *p* < 0.001), with private insurance (65.4% vs. 52.4%, *p* < 0.001), and fewer comorbidities (CD0; 82.3% vs. 76.9%, *p* < 0.001) were more likely to receive adjuvant radiotherapy (Table S1).

### 3.2. The Presence of Any Individual Adverse Feature Is Independently Associated with Survival in HPV-OPC

**Figure 2:**
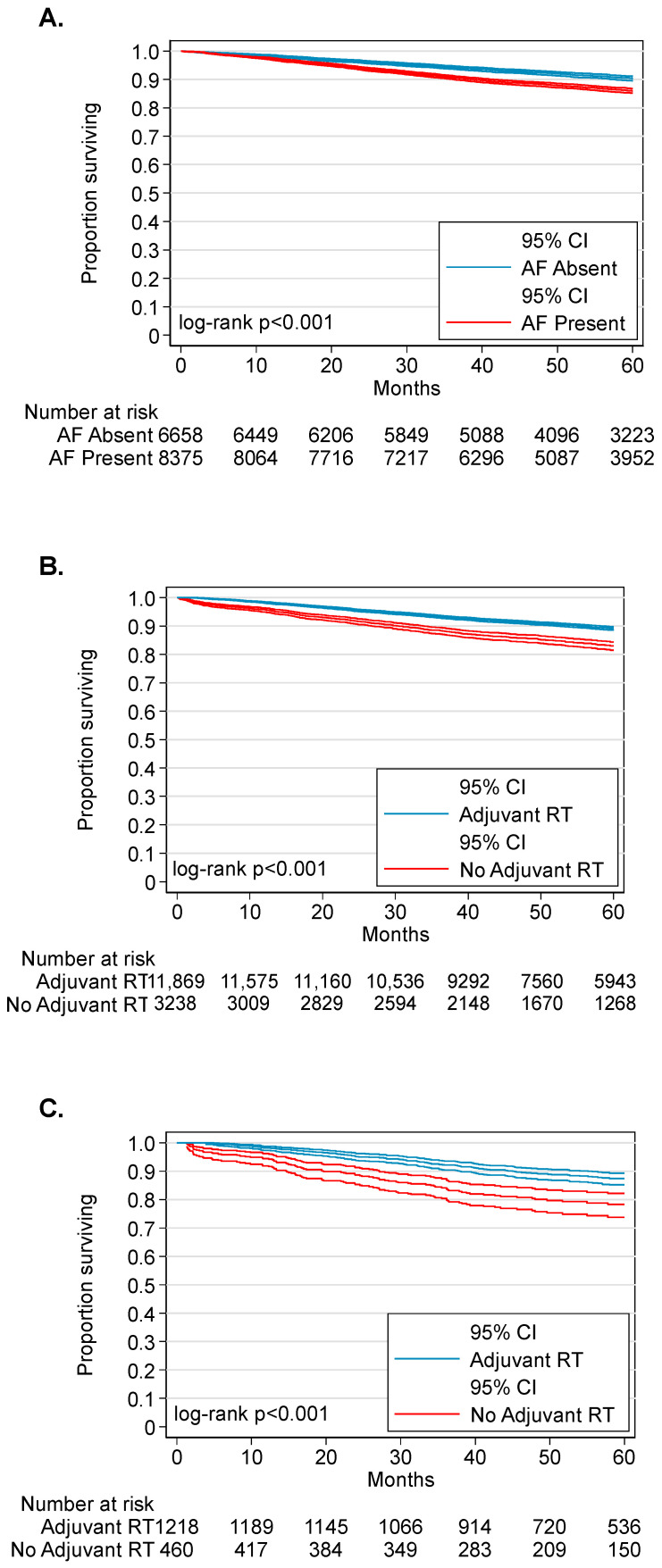
Kaplan–Meier Analyses (**A**) Influence of Adverse Features on Survival in HPV-OPC (**B**) Influence of Adjuvant RT on Survival in HPV-OPC (**C**) Influence of Adjuvant RT on Survival in AF-HPV-OPC.

Survival differences by adverse feature were examined. The median follow-up time for the study population was 58.4 months (interquartile range, 40.4–80.1). Three-year overall survival was 92.3% (95%CI, 91.9–92.7%) and 5-year overall survival was 87.9% (95%CI, 87.3–88.5%). Factors associated with survival for the overall cohort are shown in Table 2 and Table S2. On unadjusted analysis, increasing age, Black race, primary tumor subsite other than tonsil or base of tongue, T2 tumor category, N3 nodal category, the presence of any AF, each individual adverse feature, and comorbidity score were associated with poor survival. The presence of any AF diminished median OS at 3 years (90.8% [95%CI 90.1–91.4] vs. 94.3% [95%CI 93.7–94.8], *p* < 0.001; Figure 2A). Receipt of radiotherapy, private insurance status, and residences in upper income quartile ZIP code were associated with improved survival.

Adjustment for factors associated with survival in unadjusted Cox models demonstrated that the presence of any AF was independently associated with worse survival (aHR_anyAF_ 1.56, 95%CI 1.40–1.73, *p* < 0.001). To determine the contribution of each individual adverse feature to survival, adjusted analyses were performed for each AF in isolation. These analyses found PSM (aHR_PSM_ 1.57, 95%CI 1.41–1.75, *p* < 0.001), ENE (aHR_ENE_ 1.74, 95% CI 1.52–2.00, *p* < 0.001), LVI (aHR_LVI_ 1.45, 95% CI 1.29–1.64, *p* < 0.001), and LN4/5 (aHR_LN4/5_ 1.63, 95% CI 1.42–1.86, *p* < 0.001) each to be independently associated with worse survival (Table 2: “Total HPV-OPC Cohort” and Table S2).

### 3.3. Adjuvant Radiotherapy Is Associated with Improved Survival in Patients with AF-HPV-OPC

In patients with HPV-OPC, receipt of adjuvant radiotherapy was associated with 4.8% improvement in median OS at 3 years (93.3%, 95%CI 92.9–93.7 vs. 88.5%, 95%CI 87.3–89.6, *p* < 0.001; Figure 2B). To evaluate the prognostic effect of adjuvant radiotherapy on AF, Cox proportional hazards models were applied to patients with AF-HPV-OPC (n = 8375). Similar to the full cohort unadjusted analysis showed increasing age, Black race, primary tumor subsite other than tonsil or base of tongue, T2 tumor category, each individual AF, and comorbidity score to be associated with poor survival and receipt of radiotherapy, private insurance status, and residence in upper income quartile ZIP codes to be associated with improved survival in the AF-HPV-OPC stratum. Adjustment for these factors showed that adjuvant radiotherapy was independently associated with improved survival for patients with any AF-HPV-OPC and conferred a 40% reduction in the risk of death (aHRAF 0.60, 95% CI 0.51–0.69, *p* < 0.001). Adjusted analyses performed for each AF in isolation showed that adjuvant radiotherapy reduced the risk of death by 40–44% for each individual AF (Table 2: “AF-HPV-OPC Cohort” and Table S3).

To further mitigate selection bias in evaluating the effect of adjuvant radiotherapy on AF, patients with AF-HPV-OPC were stratified into propensity score matched (PM) cohorts who received (Rad+, n = 1218) or did not receive (Rad−, n = 460) adjuvant radiotherapy. PM resulted in balanced distribution of baseline variables except Rad+ patients had a higher proportion of N1 (86.4% vs. 75.9%, *p* < 0.001, Table S4 and Figure S2). In the PM cohort, associations between AFs were diminished or revealed negative correlations with other AFs (Figure 1B). After PM, receipt of adjuvant radiotherapy was associated with a more pronounced 9.9% improvement in median OS at 3 years (3-year OS: 92.0%, 95%CI 91.3–92.7 vs. 82.1%, 95%CI 79.6–84.3, *p* < 0.001; Figure 2C). In the PM cohort, unadjusted analysis found increasing age, T2 tumor category, PSM, ENE, and presence of comorbidities to be associated with worse survival. Receipt of adjuvant radiotherapy, private insurance status, and residence in upper quartile income ZIP codes were associated with improved survival. Adjustment for these factors showed T2 tumor category, uninsured status, government insurance, and increased comorbidity score to be independently associated with poor survival (Table 3). While ENE (aHR 1.75, 95%CI 1.35–2.28, *p* < 0.001) and PSM (aHR 1.58, 95% 1.19–2.10, *p* = 0.002) were independently associated with poor survival, LVI and LN4/5 were not significantly associated with survival. Adjuvant radiation conferred improved survival (aHR 0.55, 95% 0.43–0.71, *p* < 0.001).

### 3.4. Trends of AF-HPV-OPC Treated with Adjuvant Radiation

**Figure 3:**
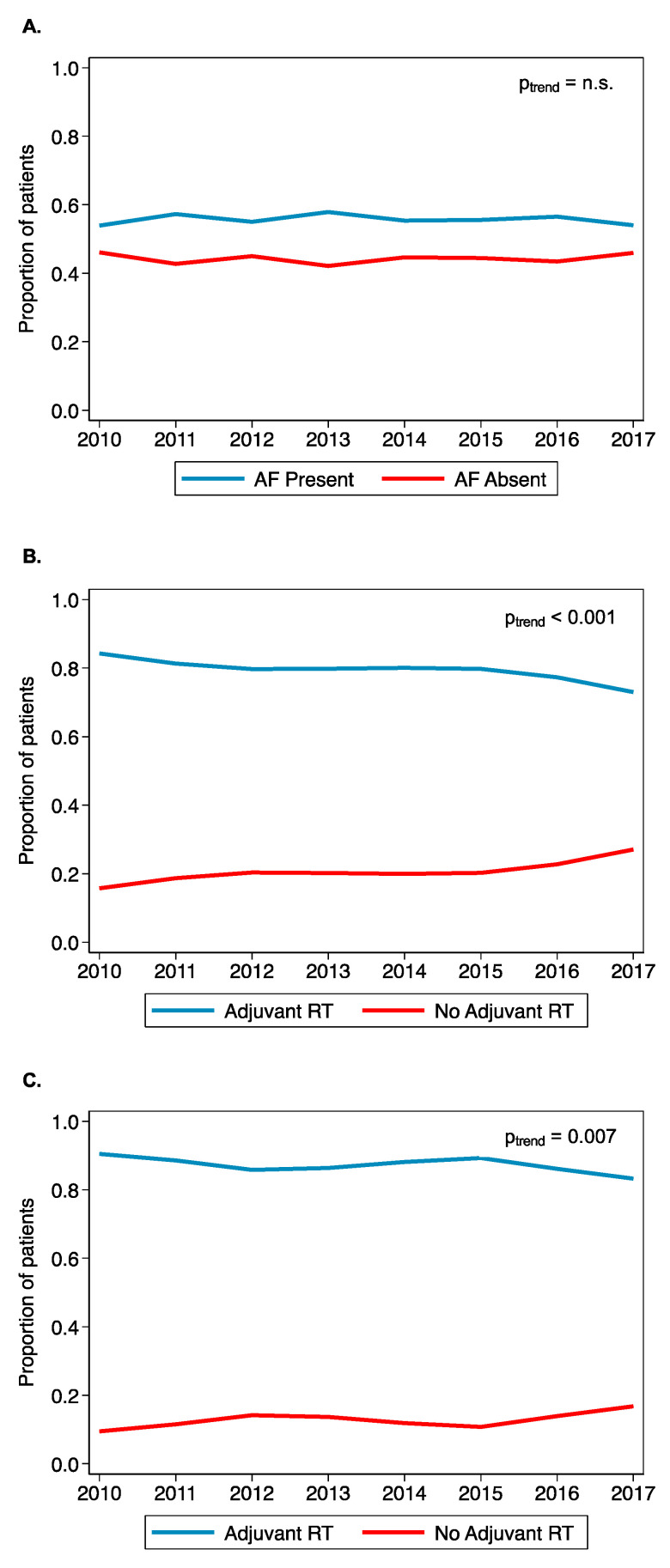
Trends in Adjuvant Radiotherapy and Adverse Features in HPV-OPC (**A**) Trends in HPV-OPC with Adverse Features (**B**) Trends in HPV-OPC receiving Adjuvant RT (**C**) Trends in AF-HPV-OPC receiving Adjuvant RT.

Across the study period, the proportion of AF-positive tumors remained constant (p_trend_ = 0.37; Figure 3A). The proportion of HPV-OPC treated with adjuvant radiotherapy declined from 80.1% in 2014 to 73.0% in 2017 (p_trend_ < 0.001; Figure 3B). Overall, 13.2% of patients with AF-HPV-OPC did not receive adjuvant radiotherapy. The fraction of AF-HPV-OPC treated with adjuvant radiotherapy declined from 90.5% in 2010 to a nadir of 83.2% in 2017 (p_trend_ = 0.007) (Figure 3C).

## 4. Discussion

In this study, we show that adjuvant radiotherapy was associated with improved OS for patients with early-stage HPV-OPC found to have AF following primary surgery. Specifically, we found that uninsured status, government insurance, T2 primary tumor category, Charlson-Deyo comorbidity score, ENE, and PSM were associated with worse survival. Adjuvant radiotherapy was associated with 45% reduction in risk of death in adjusted Cox analysis of the propensity score matched cohort. These findings suggest that adjuvant radiation may improve oncologic outcomes in early-stage HPV-OPC positive for AF.

Current NCCN guidelines recommend adjuvant therapy for surgically treated HPV-OPC demonstrating AF, including the high-risk features ENE and PSM. However, the histopathologic indications for adjuvant therapy in HPV-OPC are largely based on prospective trials performed in patients with HPV-negative head and neck squamous cell carcinoma [17,29,30,31], and have fueled debate on the prognostic importance of AF in HPV-OPC. Indeed, multiple retrospective studies have suggested that some AF may not be associated with outcomes in HPV-OPC [14,19,20,21,22,32,33,34]. In a retrospective analysis of 106 patients, Iyer and colleagues showed PSM, ENE, and LVI did not associate with survival in HPV-OPC [32]. Similarly, in a cohort of 220 patients with HPV-OPC Sinha et al. revealed that ENE was not prognostic for disease-specific survival (*p* = 0.85) or disease-free survival (*p* = 0.62) [33] and Nichols et al. in a retrospective analysis of 48 patients with HPV-OPC revealed data that suggested PSM and ENE in patients treated with primary transoral robotic surgery (TORS) may not affect outcomes [19]. Many of these studies are limited by sample size and short follow-up durations. Our PM analysis of AF-HPV-OPC revealed that adverse features ENE (HR = 1.81, *p* < 0.001) and PSM (HR = 1.58, *p* = 0.002) were most strongly associated with increased risk of death. Furthermore, we demonstrate that adjuvant radiation is associated with a nearly 10% lower risk of death at 3 years in patients with AF-positive HPV-OPC (92.0% vs. 82.1%, *p* < 0.001, Figure 2C).

Pathologic AFs are the downstream characteristics of what is undoubtedly a complex web of genetic and epigenetic interactions. It is not surprising that AFs tend to co-localize. “Intermediate” adverse features such as LVI and LN4/5 often co-occur with high-risk features of ENE and PSM. In our analysis 45.7% of patients with LVI and 48.9% of patients with LN4/5 had co-occurrence of ENE (LVI: 1013 of 2809; LN4/5: 652 of 1710) or PSM (LVI: 809 of 2809; LN4/5: 528 of 1710). Given that AF often co-occur, the presence of multiple AF may be associated with worse outcomes. After PM, our cohort accounts for this by diminishing associations between AFs and then assess their association with outcomes in HPV-OPC (Figure 1). Recent efforts have focused on re-stratifying traditional guideline-based risks to account for their summative risk on oncologic outcomes. Cramer and colleagues designed and assessed a novel composite risk score that stratified patients into 3 risk groups which predict significantly different outcomes based on pathological risk (5-year OS for low-, intermediate-, and high-risk: 76.2% vs. 54.5% vs. 40.9%) [15]. This analysis showed that microscopic (HR 1.66, 95%CI 1.18–2.32) and macroscopic ENE (HR 2.20, 95%CI 1.28–3.75) as well as LVI (HR 1.54, 95%CI 1.15–2.06) were associated with poor survival, however, LN4/5 and PSM were not prognostically significant in HPV-OPC on multivariate analyses. Despite these findings, their risk stratification score weighted PSM and ENE more heavily than other AF and revealed that LN4/5 was not predictive of poor outcomes. Consistent with these findings, we demonstrate that the presence of PSM and ENE but not LN4/5 were associated with poor survival. By contrast, LVI was not prognostic of outcomes in our PM analysis. Taken together, the traditional pathologic risk stratification system for HPV-OPC may not be representative of outcomes, and importantly, intermediate adverse features LVI and LN4/5 may not represent strong prognostic features in the context of HPV-OPC.

Therapeutic de-intensification for early-stage HPV-OPC is being increasingly investigated [2,17,35]. However, capabilities of selecting patients for de-escalation remain in their infancy. Emerging prospective investigations from the ECOG-ACRIN 3311 trial (E3311) demonstrated greater than 90% 2-year progression free survival utilizing adverse feature risk stratification. Ongoing trials are attempting to determine the allocation of adjuvant therapy based on AF (LVI, LN4/5, ENE, and PSM status) including the SIRS (NCT02072148) and MINT trials (NCT03621696) [9,36]. However, several retrospective studies have postulated that omitting adjuvant radiotherapy in early-stage HPV-OPC with positive AF may not affect oncologic outcomes [19,21]. In a retrospective cohort of 364 patients with HPV-OPC treated with TORS, adjuvant radiotherapy was not associated with an improvement in survival among patients with clinicopathologic indications for adjuvant radiation, including PSM, ENE, and LN4/5. However, patients in the study who did not receive adjuvant radiotherapy showed 8-fold greater locoregional failure rates at 3 years (32% vs. 4%, *p* < 0.001) [37].

Our data showed a downtrend in the use of adjuvant therapy despite stable incidence of AFs. We are unable to ascertain the reasons behind these national practice patterns, but they are undoubtedly multifactorial. National guidelines for adjuvant therapy remained unchanged across the study period. Declining rates of adjuvant radiotherapy for AF-HPV-OPC may suggest changes in practice patterns despite current guidelines that recommend adjuvant radiotherapy [38,39]. The present study supports the notion that, until emerging de-escalation data from ongoing clinical trials determine the allocation or omission of adjuvant therapy based on AF, patients with AF-HPV-OPC should receive adjuvant radiation as instructed by NCCN guidelines. Although any AF was associated with worse survival (HR = 1.56, 95% CI 1.41–1.73, *p* < 0.001), we demonstrate that in a propensity matched score cohort, intermediate-risk features may be less prognostic for outcomes and warrant investigation for possible de-intensification paradigms.

Limitations in our study include its retrospective design and lack of standardized follow-up protocols. Furthermore, the NCDB lacks cause-specific outcomes data which may confound analyses of treatment effects in observational studies [40]. Importantly, the NCDB lacks data on a well described adverse feature, perineural invasion, as well as other pathologic risk factors such as close margins and other factors associated with survival, including tobacco use status and cumulative lifetime tobacco use, and therefore our analyses were unable to incorporate these potentially important prognostic indicators. Re-resection of positive surgical margins represents one potential factor that may contribute to overestimating rates of AF-HPV-OPC patients who did not receive adjuvant radiotherapy. Unfortunately, limitations in NCDB precluded evaluating patients with PSM-positive disease who underwent re-resection. Adjuvant radiation dosing and patients who received adjuvant chemotherapy were not assessed in our analyses which may impact outcomes reported. Indeed, we confined analyses to patients who may be most amenable to treatment de-intensification, including tumors that only demonstrated intermediate risk features, which a priori tends to exclude patients who would require chemotherapy. However, our findings represent a large cohort of patients and utilizes validated propensity score matching analyses that may accurately capture significant factors predictive for the outcomes assessed in this study. Nevertheless, propensity score matching analyses do not account for potential unmeasured confounding variables and conclusions from these analyses require further investigation using randomized controlled studies.

## 5. Conclusions

Results from this national database analysis indicate that adjuvant radiation significantly prolongs survival in surgically treated HPV-OPC positive for AF, however, nearly 13% of these patients do not receive adjuvant radiotherapy. Our study supports adherence to established NCCN-recommended guidelines for adjuvant therapy. Emerging results from de-escalation trials will be welcomed contributions to our understanding of allocation or omission of adjuvant therapy, and should be carefully considered.

## Figures and Tables

**Table 1:** Baseline Characteristics by Adverse Feature Status.

Variable		Overall *n* = 15,036	AF Negative *n* = 6661	AF Positive *n* = 8375	*p*-Value
**Age** (mean, SD)		58.3(9.4)	58.3 (9.3)	58.3 (9.4)	0.858
**Sex**	Male	12,564	5484 (82.3)	7080 (84.5)	<0.001
**Race**	White	13,790	6067 (91.1)	7723 (92.2)	0.036
	Black	525	240 (3.6)	285 (3.4)	
	Hispanic	364	186 (2.8)	178 (2.1)	
	Asian/Pacific Islander	133	68 (1.0)	65 (0.8)	
**Primary site**	Tonsil	10,768	4689 (70.4)	6079 (72.6)	0.002
	Base of tongue	3548	1616 (24.3)	1932 (23.1)	
	Other oropharynx	720	356 (5.3)	364 (4.4)	
**Tumor Category**	T1	8116	3797 (57.0)	4319 (51.6)	<0.001
	T2	6920	2864 (43.0)	4056 (48.4)	
**Nodal Category**	N0	2577	1768 (26.5)	809 (9.7)	<0.001
	N1	11,331	4576 (68.7)	6755 (80.7)	
	N2	599	178 (2.7)	421 (5.0)	
	N3	437	97 (1.5)	340 (4.1)	
**Tumor Margin Status**	Negative	9640	5657 (84.9)	3983 (47.6)	<0.001
	Positive	4033	0 (0.0)	4033 (48.2)	
	Unknown	1363	1004 (15.1)	359 (4.3)	
**Lymphovascular Invasion**	Negative	8556	4709 (70.7)	3847 (45.9)	<0.001
	Positive	2809	0 (0.0)	2809 (33.5)	
	Unknown	3671	1952 (29.3)	1719 (20.5)	
**Extranodal Extension**	Negative	6543	4090 (61.4)	2453 (29.3)	<0.001
	Positive	3238	0 (0.0)	3238 (38.7)	
	Unknown	5255	2571 (38.6)	2684 (32.1)	
**Level 4/5 Lymph Node**	Negative	12,571	6347 (95.3)	6224 (74.3)	<0.001
	Positive	1710	0 (0.0)	1710 (20.4)	
	Unknown	755	314 (4.7)	441 (5.3)	
**Adjuvant Radiotherapy**	Yes	11,804	4537 (68.1)	7267 (86.8)	<0.001
**Charlson-Deyo Comorbidity Score**	0	12,355	5519 (82.9)	6836 (81.6)	0.107
	1	2021	874 (13.1)	1147 (13.7)	
	2	432	181 (2.7)	251 (3.0)	
	3	228	87 (1.3)	141 (1.7)	
**Primary Payor**	Not Insured	323	137 (2.1)	186 (2.2)	0.280
	Private	9625	4295 (64.5)	5330 (63.6)	
	Medicaid/Medicare	4904	2138 (32.1)	2766 (33.0)	
**Median Household Income**	>$63,000	5340	2452 (42.5)	2888 (40.0)	0.031
	$48,000–62,999	3536	1550 (26.9)	1986 (27.5)	
	$38,000–47,999	2608	1115 (19.3)	1493 (20.7)	
	<$38,000	1500	651 (11.3)	849 (11.8)	

**Table 2:** Cox Proportional Hazards Models of Adverse Features.

	Unadjusted Cox Analysis			Adjusted Cox Analyses														
	HR	95% CI	*p*-Value	aHR_AF_	95% CI	*p*-Value	aHR_PSM_	95% CI	*p*-Value	aHR_ENE_	95% CI	*p*-Value	aHR_LVI_	95% CI	*p*-Value	aHR_LN4/5_	95% CI	*p*-Value
**Total HPV-OPC Cohort**			***n* = 15,036**			***n* = 12,780**			***n* = 12,780**			***n* = 12,780**			***n* = 12,780**			***n* = 12,780**
**Any AF**																		
No	1.00	--	--	1.00	--	--												
Yes	1.44	1.31–1.58	<0.001	1.56	1.40–1.73	<0.001												
**PSM**																		
Negative	1.00	--	--				1.00	--	--									
Positive	1.49	1.35–1.64	<0.001				1.57	1.41–1.75	<0.001									
**ENE**																		
Negative	1.00	--	--							1.00	--	--						
Positive	1.79	1.59–2.01	<0.001							1.74	1.52–2.00	<0.001						
**LVI**																		
Negative	1.00	--	--										1.00	--	--			
Positive	1.54	1.38–1.71	<0.001										1.45	1.29–1.64	<0.001			
**LN4/5**																		
Negative	1.00	--	--													1.00	--	--
Positive	1.63	1.44–1.83	<0.001													1.63	1.42–1.86	<0.001
**AF-HPV-OPC Cohort**			***n* = 8375**			***n* = 7103**			***n* = 7103**			***n* = 7103**			***n* = 7103**			***n* = 7103**
**Adjuvant RT**																		
No	1.00	--	--	1.00	--	--	1.00	--	--	1.00	--	--	1.00	--	--	1.00	--	--
Yes	0.48	0.42–0.55	<0.001	0.60	0.51–0.69	<0.001	0.56	0.49–0.66	<0.001	0.56	0.48–0.65	<0.001	0.60	0.52–0.70	<0.001	0.60	0.52–0.69	<0.001

Abbreviations: AF, Adverse Features; RT, Radiotherapy; PSM, Positive surgical margins; ENE, Extranodal extension; LVI, Lymphovascular invasion; LN4/5, level 4/5 positive cervical lymph node.

**Table 3:** Cox Proportional Hazards Analysis of the Propensity Score Matched Cohort.

		Unadjusted (n = 1678)			Adjusted (n = 1678)		
		HR	95% CI	*p*-Value	HR	95% CI	*p*-Value
**Age**		1.040	1.026–1.053	<0.001	1.014	0.999–1.030	0.064
**Race**	White	1.000	--	--			
	Black	1.509	0.945–2.408	0.085			
	Hispanic	0.748	0.278–2.009	0.564			
	Asian/Pacific Islander	1.737	0.431–6.992	0.437			
**Primary site**	Tonsil	1.000	--	--			
	Base of tongue	1.120	0.863–1.454	0.395			
	Other oropharynx	1.539	0.944–2.509	0.084			
**Tumor Category**	T1	1.000	--	--	1.000	--	--
	T2	1.607	1.255–2.057	<0.001	1.425	1.105–1.838	0.006
**Nodal Category**	N0	1.000	--	--			
	N1	0.890	0.611–1.297	0.544			
	N2	1.203	0.553–2.618	0.641			
	N3	0.807	0.371–1.755	0.588			
**Tumor Margin Status**	Negative	1.000	--	--	1.000	--	--
	Positive	1.308	1.003–1.705	0.048	1.581	1.188–2.103	0.002
**Lymphovascular Invasion**	Negative	1.000	--	--	1.000	--	--
	Positive	1.010	0.791–1.288	0.939	1.241	0.959–1.608	0.101
**Extranodal Extension**	Negative	1.000	--	--	1.000	--	--
	Positive	1.701	1.333–2.171	<0.001	1.753	1.348–2.279	<0.001
**Level 4/5 Lymph Node**	Negative	1.000	--	--	1.000	--	--
	Positive	1.184	0.895–1.566	0.237	1.266	0.954–1.681	0.103
**Adjuvant Radiotherapy**	No	1.000	--	--	1.000	--	--
	Yes	0.548	0.425–0.705	<0.001	0.552	0.428–0.713	<0.001
**Charlson-Deyo Comorbidity Score**	0	1.000	--	--	1.000	--	--
	1	1.701	1.270–2.277	<0.001	1.442	1.073–1.937	0.015
	2	1.645	0.841–3.218	0.146	1.277	0.648–2.516	0.481
	3	3.972	2.544–6.203	<0.001	2.723	1.727–4.294	<0.001
**Primary Payor**	Private insurance	1.000	--	--	1.000	--	--
	Medicare/Medicaid/Gov.	2.716	2.065–3.571	<0.001	2.083	1.521–2.851	<0.001
	Not insured	2.884	1.566–5.313	0.001	2.971	1.605–5.502	0.001
**Median Household Income**	>$63,000	1.000	--	--			
	$48,000–$62,999	1.409	1.014–1.957	0.041			
	$38,000–$47,999	1.325	0.934–1.880	0.115			
	<$38,000	1.988	1.392–2.838	<0.001			

## Data Availability

Research data were acquired by application to participant user data files in accordance with the American College of Surgeons Commission on Cancer: https://ncdbapp.facs.org/puf/ accessed on 3 March 2022.

## Supplementary Materials

The following supporting information can be downloaded at: https://www.mdpi.com/article/10.3390/cancers14184515/s1, Figure S1: Patient Inclusion and Analytic Approach; Figure S2: Propensity Score Matching Plot; Table S1: Baseline Characteristics by Adjuvant Radiotherapy; Table S2: Cox Proportional Hazards Analysis of Patients with HPV-OPC; Table S3: Cox Proportional Hazards Analysis in Patients with AF-positive HPV-OPC; Table S4: Baseline Characteristics of the Propensity Score Matched Cohort.
