# Supplementary material for: Adjuvant Radiotherapy in Surgically Treated HPV-Positive Oropharyngeal Carcinoma with Adverse Pathological Features
Source: Cancers (Basel). 2022 Sep 17;14(18):4515. doi: 10.3390/cancers14184515 (PMC9496867; doi:10.3390/cancers14184515)

Table S1: Baseline Characteristics by Adjuvant Radiotherapy

Variable	Overall n = 8,375	Did Not Receive Adjuvant Radiotherapy n = 1,108	Received Adjuvant Radiotherapy n = 7,267	p-value
**Age** (mean, SD)	8,375	60.9 (10.4)	57.9 (9.1)	<0.001
**Sex**				0.258
Male	7,080	924 (83.4)	6,156 (84.7)	
Female	1,295	184 (16.6)	1,111 (15.3)	
**Race**				0.068
White	7,723	1,009 (91.1)	6,714 (92.4)	
Black	285	45 (4.1)	240 (3.3)	
Hispanic	178	24 (2.2)	154 (2.1)	
Asian/Pacific Islander	65	5 (0.5)	60 (0.8)	
**Primary site**				<0.001
Tonsil	6,079	714 (64.4)	5,365 (73.8)	
Base of tongue	1,932	318 (28.7)	1,614 (22.2)	
Other oropharynx	364	76 (6.9)	288 (4.0)	
**Tumor Category**				0.416
T1	4,319	584 (52.7)	3,735 (51.4)	
T2	4,056	524 (47.3)	3,532 (48.6)	
**Nodal Category**				<0.001
N0	809	215 (19.4)	594 (8.2)	
N1	6,755	797 (71.9)	5,958 (82.0)	
N2	421	36 (3.3)	385 (5.3)	
N3	340	48 (4.3)	292 (4.0)	
**Tumor Margin Status**				<0.001
Negative	3,983	651 (58.8)	3,332 (45.9)	
Positive	4,033	430 (38.8)	3,603 (49.6)	
Unknown	359	27 (2.4)	332 (4.6)	
**Lymphovascular Invasion**				<0.001
Negative	3,847	462 (41.7)	3,385 (46.6)	
Positive	2,809	489 (44.1)	2,320 (31.9)	
Unknown	1,719	157 (14.2)	1,562 (21.5)	
**Extranodal Extension**				<0.001
Negative	2,453	464 (41.9)	1,989 (27.4)	
Positive	3,238	386 (34.8)	2,852 (39.3)	
Unknown	2,684	258 (23.3)	2,426 (33.4)	
**Level 4/5 Lymph Node**				0.024
Negative	6,224	841 (75.9)	5,383 (74.1)	
Positive	1,710	197 (17.8)	1,513 (20.8)	
Unknown	441	70 (6.3)	371 (5.1)	
**Charlson-Deyo Comorbidity Score**				<0.001
0	6,836	852 (76.9)	5,984 (82.3)	
1	1,147	174 (15.7)	973 (13.4)	
2	251	47 (4.2)	204 (2.8)	
3	141	35 (3.2)	106 (1.5)	
**Primary Payor**				<0.001
Not Insured	186	33 (3.0)	153 (2.1)	
Private	5,330	581 (52.4)	4,749 (65.4)	
Medicaid / Medicare / Gov.	2,766	479 (43.2)	2,287 (31.5)	
**Median Household Income**				0.122
> $63,000	2,888	366 (37.2)	2,522 (40.5)	
$48,000 - 62,999	1,986	275 (28.0)	1,711 (27.5)	
$38,000 - 47,999	1,493	208 (21.2)	1,285 (20.6)	
< $38,000	849	134 (13.6)	715 (11.5)	

Table S2: Cox Proportional Hazards Analysis of Patients with HPV-OPC

		Unadjusted Analysis			Adjusted Analyses														
		n=15,036			Any AF (n=12,780)			PSM (n=12,780)			ENE (n=12,780)			LVI (n=12,780)			LN4/5 (n=12,780)		
		HR	95% CI	p-value	aHR_{AF}	95% CI	p-value	aHR_{PSM}	95% CI	p-value	aHR_{ENE}	95% CI	p-value	aHR_{LVI}	95% CI	p-value	$aHR_{LN4/5}$	95% CI	p-value
**Age**		1.057	1.052-1.062	<0.001	1.036	1.030-1.043	<0.001	1.037	1.031-1.043	<0.001	1.036	1.030-1.042	<0.001	1.037	1.031-1.043	<0.001	1.037	1.031-1.043	<0.001
**Sex**	Male	1.000	--	--															
	Female	0.982	0.872-1.105	0.760															
**Race**	White	1.000	--	--	1.000	--	--	1.000	--	--	1.000	--	--	1.000	--	--	1.000	--	--
	Black	1.712	1.412-2.075	<0.001	1.418	1.148-1.751	0.001	1.416	1.149-1.746	0.001	1.389	1.127-1.713	0.002	1.402	1.138-1.729	0.002	1.425	1.156-1.757	0.001
	Hispanic	0.696	0.488-0.992	0.045	0.709	0.484-1.038	0.077	0.683	0.466-1.000	0.050	0.684	0.467-1.002	0.051	0.667	0.456-0.977	0.038	0.689	0.470-1.009	0.055
	Asian/Pacific Islander	1.169	0.753-1.817	0.487	1.341	0 .831-2.165	0.230	1.303	0.818-2.077	0.265	1.297	0.814-2.067	0.274	1.276	0.801-2.032	0.305	1.282	0.805-2.042	0.296
**Primary site**	Tonsil	1.000	--	--	1.000	--	--	1.000	--	--	1.000	--	--	1.000	--	--	1.000	--	--
	Base of tongue	1.014	0.913-1.127	0.791	0.868	0.772-0.976	0.018	0.904	0.804-1.016	0.090	0.902	0.802-1.015	0.086	0.863	0.768-0.969	0.013	0.854	0.760-0.959	0.008
	Other oropharynx	1.706	1.432-2.032	<0.001	1.336	1.107-1.611	0.002	1.359	1.127-1.639	0.001	1.278	1.059-1.542	0.010	1.323	1.098-1.595	0.003	1.315	1.091-1.585	0.004
**Tumor category**	T1	1.000	--	--	1.000	--	--	1.000	--	--	1.000	--	--	1.000	--	--	1.000	--	--
	T2	1.400	1.282-1.529	<0.001	1.287	1.169-1.417	<0.001	1.286	1.168-1.416	<0.001	1.313	1.193-1.445	<0.001	1.290	1.172-1.420	<0.001	1.313	1.193-1.445	<0.001
**Nodal category**	N0	1.000	--	--	1.000	--	--	1.000	--	--	1.000	--	--	1.000	--	--	1.000	--	--
	N1	0.671	0.602-0.748	<0.001	0.783	0.691-0.888	<0.001	0.882	0.780-0.998	0.046	0.914	0.802-1.042	0.179	0.822	0.725-0.931	0.002	0.796	0.701-0.903	<0.001
	N2	1.208	0.989-1.475	0.064	1.468	1.170-1.843	0.001	1.606	1.284-2.007	<0.001	1.580	1.263-1.978	<0.001	1.561	1.248-1.953	<0.001	1.423	1.132-1.789	0.003
	N3	1.327	1.059-1.661	0.014	1.383	1.074-1.780	0.012	1.765	1.381-2.257	<0.001	1.677	1.299-2.165	<0.001	1.576	1.231-2.019	<0.001	1.432	1.113-1.843	0.005
	Unknown	1.178	0.725-1.912	0.509	1.036	0.582-1.843	0.905	1.069	0.614-1.861	0.815	0.972	0.558-1.693	0.921	1.169	0.671-2.034	0.582	1.095	0.627-1.911	0.751
**Adverse features**	No	1.000	--	--	1.000	--	--												
	Yes	1.438	1.312-1.576	<0.001	1.559	1.404-1.732	<0.001												
**PSM**	Negative	1.000	--	--				1.000	--	--									
	Positive	1.490	1.354-1.640	<0.001				1.572	1.412-1.750	<0.001									
	Unknown	1.291	1.116-1.494	0.001				1.270	1.081-1.491	0.004									
**ENE**	Negative	1.000	--	--							1.000	--	--						
	Positive	1.785	1.585-2.010	<0.001							1.744	1.524-1.995	<0.001						
	Unknown	1.882	1.696-2.089	<0.001							1.844	1.639-2.074	<0.001						
**LVI**	Negative	1.000	--	--										1.000	--	--			
	Positive	1.535	1.376-1.711	<0.001										1.450	1.285-1.637	<0.001			
	Unknown	1.109	0.997-1.234	0.058										1.169	1.042-1.311	0.008			
**LN4/5**	Negative	1.000	--	--													1.000		--
	Positive	1.625	1.443-1.830	<0.001													1.626	1.422-1.861	<0.001
	Unknown	1.170	0.972-1.408	0.097													1.215	0.993-1.488	0.059
**Adjuvant radiation**	No	1.000	--	--	1.000	--	--	1.000	--	--	1.000	--	--	1.000	--	--	1.000	--	--
	Yes	0.624	0.565-0.688	<0.001	0.689	0.613-0.774	<0.001	0.673	0.598-0.757	<0.001	0.641	0.569-0.721	<0.001	0.742	0.661-0.833	<0.001	0.747	0.666-0.838	<0.001
**Charlson-Deyo Comorbidity score**	0	1.000	--	--	1.000		--	1.000	--	--	1.000	--	--	1.000	--	--	1.000	--	--
	1	1.864	1.668-2.084	<0.001	1.505	0.582-1.843	<0.001	1.505	1.334-1.698	<0.001	1.514	1.342-1.709	<0.001	1.514	1.342-1.709	<0.001	1.511	1.339-1.707	<0.001
	2	3.109	2.592-3.729	<0.001	1.953	1.598-2.387	<0.001	1.971	1.614-2.407	<0.001	1.973	1.616-2.409	<0.001	1.963	1.607-2.397	<0.001	1.978	1.620-2.415	<0.001
	3	4.866	3.930-6.024	<0.001	3.070	2.433-3.875	<0.001	3.054	2.425-3.848	<0.001	2.980	2.364-3.756	<0.001	3.005	2.384-3.788	<0.001	3.061	2.429-3.858	<0.001
**Primary payor**	Private	1.000	--	--	1.000	--	--	1.000	--	--	1.000	--	--	1.000	--	--	1.000	--	--
	Medicare/Medicaid/Gov.	2.999	2.739-3.284	<0.001	1.721	1.531-1.934	<0.001	1.715	1.526-1.927	<0.001	1.715	1.526-1.926	<0.001	1.713	1.525-1.925	<0.001	1.709	1.521-1.921	<0.001
	Not insured	2.204	1.685-2.884	<0.001	2.099	1.575-2.797	<0.001	2.053	1.540-2.736	<0.001	2.053	1.497-2.657	<0.001	2.056	1.543-2.739	<0.001	2.107	1.582-2.807	<0.001
**Median Household Income**	> 63000	1.000	--	--	1.000	--	--	1.000	--	--	1.000	--	--	1.000	--	--	1.000	--	--
	48,000 - 62,999	1.396	1.236-1.578	<0.001	1.228	1.085-1.389	0.001	1.233	1.090-1.395	0.001	1.226	1.084-1.387	0.001	1.243	1.099-1.406	0.001	1.245	1.101-1.409	<0.001
	38,000 - 47,999	1.600	1.409-1.818	<0.001	1.321	1.160-1.504	<0.001	1.311	1.152-1.492	<0.001	1.291	1.133-1.470	<0.001	1.337	1.174-1.521	<0.001	1.333	1.171-1.517	<0.001
	<38,000	2.001	1.734-2.309	<0.001	1.550	1.336-1.799	<0.001	1.551	1.337-1.799	<0.001	1.529	1.317-1.774	<0.001	1.571	1.354-1.823	<0.001	1.542	1.329-1.789	<0.001

Table S3: Cox Proportional Hazards Analysis in Patients with AF-positive HPV-OPC

		Unadjusted Analysis			Adjusted Analyses														
		n=8,375			Any AF n=7,103			PSM n=7,103			ENE n=7,103			LVI n=7,103			LN4/5 n=7,103		
		HR	95% CI	p-value	aHR_{AF}	95% CI	p-value	aHR_{PSM}	95% CI	p-value	aHR_{ENE}	95% CI	p-value	aHR_{LVI}	95% CI	p-value	$aHR_{LN4/5}$	95% CI	p-value
**Age**		1.050	1.044-1.056	<0.001	1.030	1.023-1.038	<0.001	1.030	1.023-1.038	<0.001	1.030	1.022-1.037	<0.001	1.030	1.023-1.038	<0.001	1.031	1.023-1.038	<0.001
**Sex**	Male	1.000	--	--															
	Female	1.028	0.885-1.195	0.714															
**Race**	White	1.000	--	--	1.000	--	--	1.000	--	--	1.000	--	--	1.000	--	--	1.000	--	--
	Black	1.578	1.222-2.037	<0.001	1.356	1.027-1.792	0.032	1.354	1.025-1.789	0.033	1.376	1.042-1.818	0.025	1.344	1.017-1.776	0.037	1.367	1.035-1.806	0.028
	Hispanic	0.734	0.461-1.169	0.192	0.776	0.480-1.255	0.302	0.778	0.481-1.259	0.307	0.790	0.488-1.278	0.336	0.763	0.472-1.234	0.270	0.796	0.492-1.288	0.353
	Asian/Pacific Islander	1.115	0.616-2.018	0.720	1.417	0.759-2.647	0.274	1.410	0.755-2.634	0.281	1.410	0.755-2.634	0.282	1.406	0.753-2.626	0.285	1.416	0.758-2.644	0.275
**Primary Site**	Tonsil	1.000	--	--	1.000	--	--	1.000	--	--	1.000	--	--	1.000	--	--	1.000	--	--
	Base of tongue	0.913	0.796-1.046	0.189	0.799	0.687-0.929	0.004	0.832	0.715-0.969	0.018	0.839	0.720-0.978	0.025	0.797	0.686-0.927	0.003	0.790	0.679-0.918	0.002
	Other oropharynx	1.552	1.227-1.965	<0.001	1.252	0.976-1.607	0.077	1.275	0.993-1.637	0.057	1.260	0.981-1.618	0.070	1.238	0.964-1.589	0.094	1.226	0.956-1.574	0.109
**T Stage**	T1	1.000	--	--	1.000	--	--	1.000	--	--	1.000	--	--	1.000	--	--	1.000	--	--
	T2	1.448	1.296-1.619	<0.001	1.349	1.195-1.523	<0.001	1.339	1.186-1.512	<0.001	1.334	1.182-1.505	<0.001	1.332	1.180-1.505	<0.001	1.350	1.196-1.524	<0.001
**N Stage**	N0	1.000	--	--	1.000	--	--	1.000	--	--	1.000	--	--	1.000	--	--	1.000	--	--
	N1	0.646	0.548-0.762	<0.001	0.800	0.672-0.953	0.013	0.868	0.725-1.039	0.122	0.883	0.731-1.067	0.198	0.795	0.667-0.948	0.010	0.731	0.611-0.875	0.001
	N2	1.233	0.965-1.575	0.094	1.665	1.277-2.172	<0.001	1.719	1.315-2.248	<0.001	1.709	1.305-2.237	<0.001	1.641	1.258-2.141	<0.001	1.423	1.081-1.872	0.012
	N3	1.082	0.820-1.428	0.579	1.272	0.943-1.714	0.115	1.410	1.039-1.912	0.027	1.373	1.004-1.877	0.047	1.262	0.936-1.702	0.128	1.097	0.808-1.489	0.553
	Unknown	0.773	0.363-1.647	0.505	0.594	0.220-1.605	0.304	0.569	0.211-1.538	0.267	0.528	0.195-1.427	0.208	0.617	0.228-1.669	0.342	0.563	0.208-1.526	0.259
**PSM**	Negative	1.000	--	--				1.000	--	--									
	Positive	1.284	1.145-1.439	<0.001				1.323	1.162-1.507	<0.001									
	Unknown	1.361	1.054-1.759	0.018				1.486	1.127-1.960	0.005									
**ENE**	Negative	1.000	--	--							1.000	--	--						
	Positive	1.477	1.273-1.713	<0.001							1.409	1.198-1.656	<0.001						
	Unknown	1.723	1.484-2.001	<0.001							1.680	1.423-1.983	<0.001						
**LVI**	Negative	1.000	--	--										1.000	--	--			
	Positive	1.328	1.173-1.503	<0.001										1.176	1.027-1.348	0.019			
	Unknown	1.101	0.950-1.277	0.202										1.157	0.988-1.355	0.070			
**LN4/5**	Negative	1.000	--	--													1.000	--	--
	Positive	1.438	1.266-1.632	<0.001													1.420	1.231-1.639	<0.001
	Unknown	1.296	1.029-1.632	0.027													1.235	0.953-1.599	0.111
**Adjuvant Radiation**	No	1.000	--	--	1.000	--	--	1.000	--	--	1.000	--	--	1.000	--	--	1.000	--	--
	Yes	0.484	0.422-0.554	<0.001	0.595	0.513-0.691	<0.001	0.564	0.485-0.657	<0.001	0.556	0.478-0.647	<0.001	0.602	0.518-0.700	<0.001	0.598	0.515-0.694	<0.001
**Charlson-Deyo Comorbidity Score**	0	1.000	--	--	1.000	--	--	1.000	--	--	1.000	--	--	1.000	--	--	1.000	--	--
	1	1.774	1.541-2.042	<0.001	1.422	1.220-1.657	<0.001	1.425	1.223-1.661	<0.001	1.425	1.223-1.660	<0.001	1.422	1.221-1.657	<0.001	1.424	1.222-1.660	<0.001
	2	2.793	2.212-3.525	<0.001	1.775	1.368-2.302	<0.001	1.767	1.362-2.294	<0.001	1.786	1.377-2.317	<0.001	1.762	1.358-2.286	<0.001	1.781	1.373-2.311	<0.001
	3	4.301	3.296-5.612	<0.001	2.838	2.123-3.794	<0.001	2.847	2.130-3.807	<0.001	2.868	2.145-3.837	<0.001	2.803	2.096-3.748	<0.001	2.839	2.122-3.796	<0.001
**Primary Payor**	Private	1.000	--	--	1.000	--	--	1.000	--	--	1.000	--	--	1.000	--	--	1.000	--	--
	Medicare/Medicaid/Gov.	2.821	2.518-3.160	<0.001	1.803	1.560-2.084	<0.001	1.802	1.559-2.082	<0.001	1.803	1.560-2.083	<0.001	1.800	1.557-2.081	<0.001	1.787	1.546-2.067	<0.001
	Not insured	2.419	1.748-3.348	<0.001	2.283	1.619-3.218	<0.001	2.240	1.588-3.160	<0.001	2.217	1.572-3.128	<0.001	2.258	1.601-3.184	<0.001	2.312	1.640-3.260	<0.001
**Median Household Income**	> 63000	1.000	--	--	1.000	--	--	1.000	--	--	1.000	--	--	1.000	--	--	1.000	--	--
	48,000 - 62,999	1.509	1.297-1.755	<0.001	1.343	1.153-1.565	<0.001	1.335	1.146-1.556	<0.001	1.328	1.140-1.547	<0.001	1.348	1.157-1.570	<0.001	1.348	1.157-1.570	<0.001
	38,000 - 47,999	1.566	1.332-1.841	<0.001	1.305	1.107-1.539	0.002	1.290	1.094-1.521	0.002	1.286	1.091-1.517	0.003	1.308	1.109-1.542	0.001	1.303	1.106-1.537	0.002
	<38,000	1.913	1.592-2.297	<0.001	1.479	1.224-1.788	<0.001	1.471	1.217-1.778	<0.001	1.452	1.202-1.756	<0.001	1.488	1.232-1.799	<0.001	1.453	1.202-1.756	<0.001

Table S4 - Baseline Characteristics of the Propensity Score Matched Cohort

		Overall HPV-OPC n= 1,678	No Adjuvant Radiotherapy n=460	Adjuvant Radiotherapy n=1,218	p-value
**Age** mean (SD)		61.6 (9.5)	62.5 (10.0)	61.2 (9.3)	0.011
**Race**	White	1,540 (91.8)	420 (91.3)	1,120 (92.0)	0.962
	Black	88 (5.2)	25 (5.4)	63 (5.2)	
	Hispanic	41 (2.4)	12 (2.6)	29 (2.4)	
	Asian/Pacific Islander	9 (0.5)	3 (0.7)	6 (0.5)	
**Primary site**	Tonsil	1031 (61.4)	268 (58.3)	763 (62.6)	0.254
	Base of tongue	557 (33.2)	166 (36.1)	391 (32.1)	
	Other oropharynx	90 (5.4)	26 (5.7)	64 (5.3)	
**T Stage**	T1	879 (52.4)	235 (51.1	644 (52.9)	0.513
	T2	799 (47.6)	225 (48.9)	574 (47.1)	
**N Stage**	N0	178 (10.6)	87 (18.9)	91 (7.5)	<0.001
	N1	1,401 (83.5)	349 (75.9)	1,052 (86.4)	
	N2	40 (2.4)	8 (1.7)	32 (2.6)	
	N3	59 (3.5)	16 (3.5)	43 (3.5)	
**Primary Payor**	Private insurance	813 (48.5)	210 (45.7)	603 (49.5)	0.362
	Medicare/Medicaid/Gov.	812 (48.4)	234 (50.9)	578 (47.5)	
	Not insured	53 (3.2)	16 (3.5)	37 (3.0)	
**Median Household Income**	> $63,000	564 (33.6)	143 (31.1)	421 (34.6)	0.559
	$48,000 - $62,999	477 (28.4)	138 (30.0)	339 (27.8)	
	$38,000 - $47,999	378 (22.5)	104 (22.6)	274 (22.5)	
	< $38,000	259 (15.4)	75 (16.3)	184 (15.1)	
**Charlson Deyo Score**	0	1,286 (76.6)	346 (75.2)	940 (77.2)	0.835
	1	291 (17.3)	86 (18.7)	205 (16.8)	
	2	48 (2.9)	13 (2.8)	35 (2.9)	
	3	53 (3.2)	15 (3.3)	38 (3.1)	
**Tumor Margin Status**	Negative	1,255 (74.8)	349 (75.9)	906 (74.4)	0.532
	Positive	423 (25.2)	111 (24.1)	312 (25.6)	
**Lymphovascular Invasion**	Negative	798 (47.6)	212 (46.1)	586 (48.1)	0.459
	Positive	880 (52.4)	248 (53.9)	632 (51.9)	
**Extranodal Extension**	Negative	993 (59.2)	287 (62.4)	706 (58.0)	0.100
	Positive	685 (40.8)	173 (37.6)	512 (42.0)	
**Level 4/5 Lymph Node**	Negative	1,300 (77.5)	365 (79.4)	935 (76.8)	0.259
	Positive	378 (22.5)	95 (20.7)	283 (23.2)	

Figure S1. Patient Inclusion and Analytic Approach

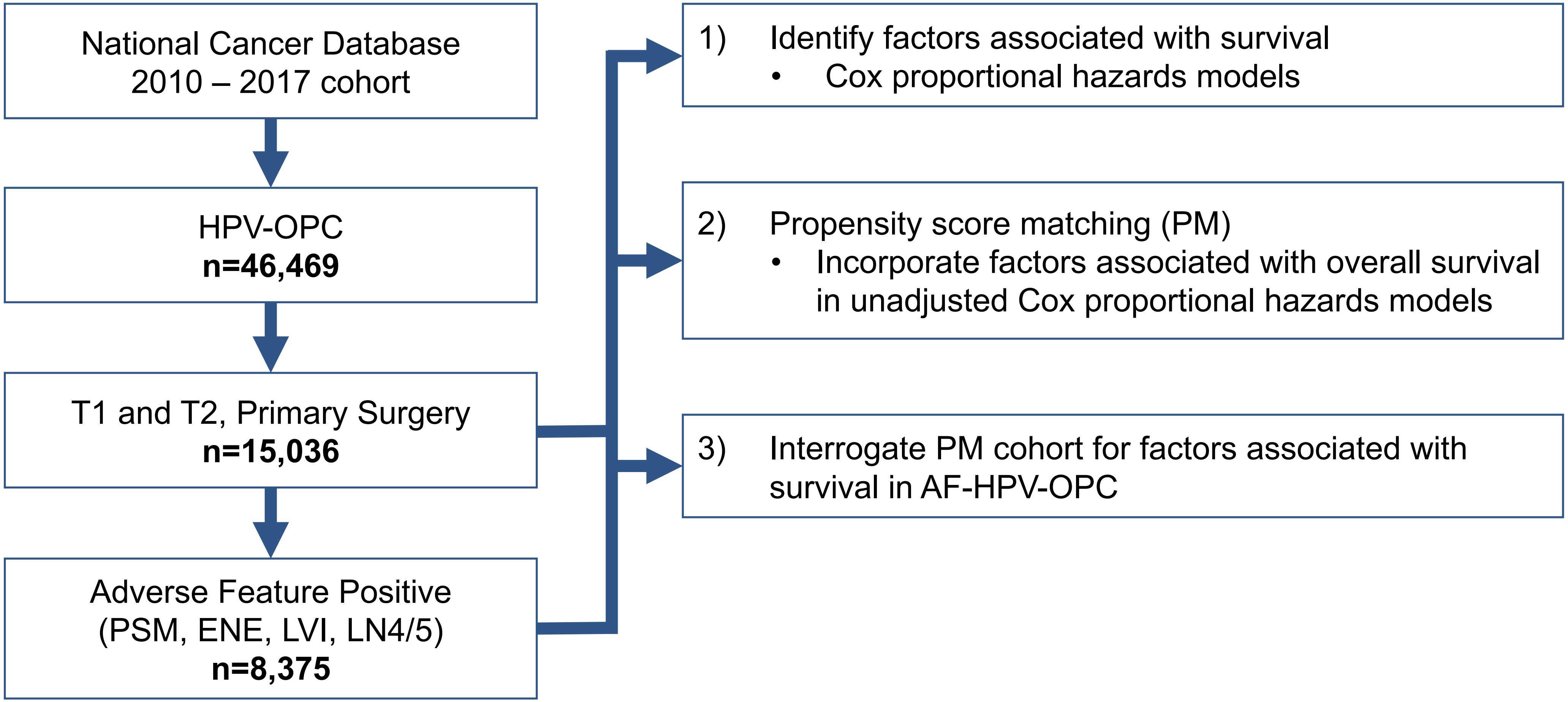

Figure S2. Propensity Score Matching Plot

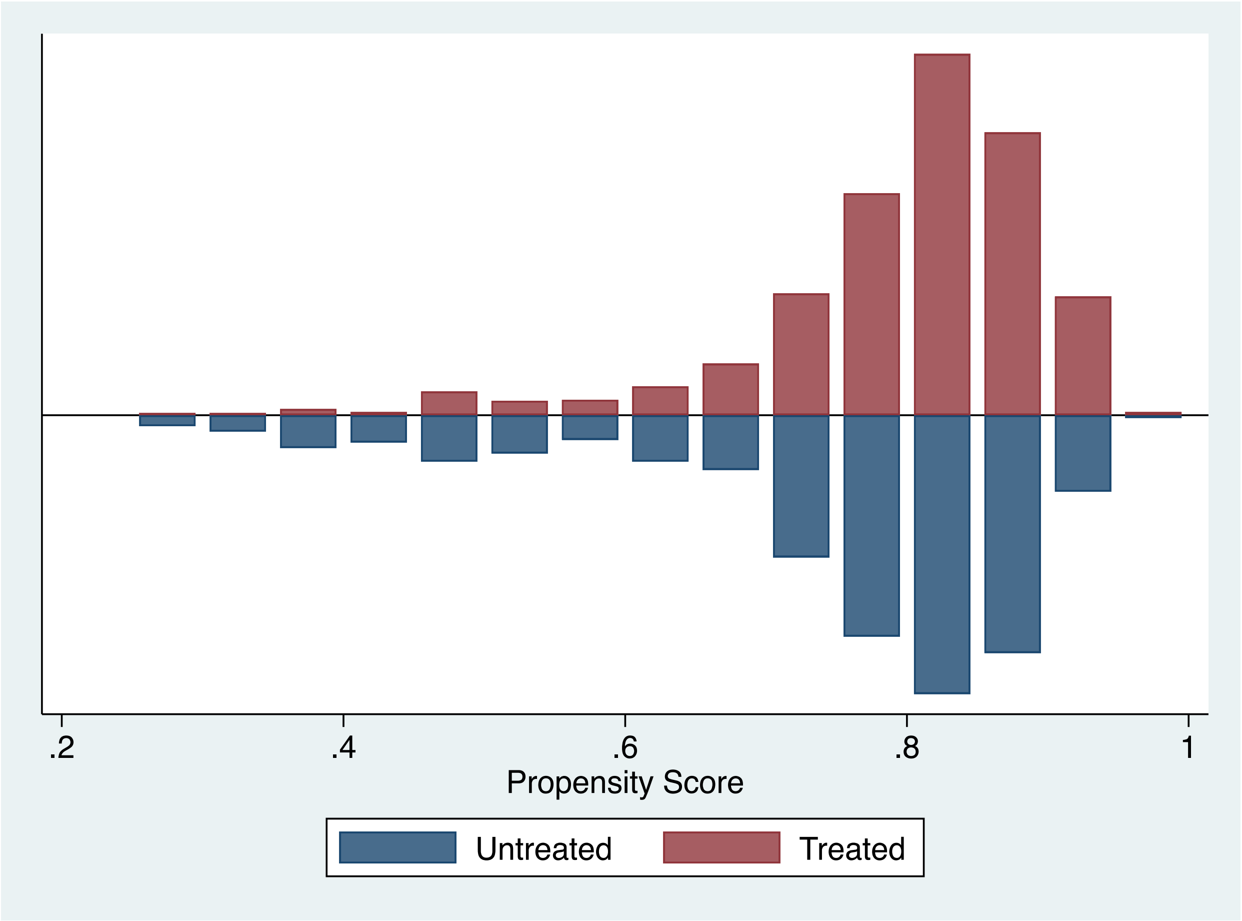

Supplement: Supplementary file 1 [file cancers-14-04515-s001.zip › cancers-1889357-supplementary.pdf]
